# Adaptation of barley to mild winters: A role for *PPDH2*

**DOI:** 10.1186/1471-2229-11-164

**Published:** 2011-11-18

**Authors:** M Cristina Casao, Ildiko Karsai, Ernesto Igartua, M Pilar Gracia, Otto Veisz, Ana M Casas

**Affiliations:** 1 Department of Genetics and Plant Production, Aula Dei Experimental Station, EEAD-CSIC, Avda. Montañana 1005, E-50059 Zaragoza, Spain; 2 Agricultural Research Institute, Hungarian Academy of Sciences, ARI-HAS, 2462 Martonvásár, Brunszvik u. 2, Hungary

## Abstract

**Background:**

Understanding the adaptation of cereals to environmental conditions is one of the key areas in which plant science can contribute to tackling challenges presented by climate change. Temperature and day length are the main environmental regulators of flowering and drivers of adaptation in temperate cereals. The major genes that control flowering time in barley in response to environmental cues are *VRNH1*, *VRNH2*, *VRNH3*, *PPDH1*, and *PPDH2 *(candidate gene *HvFT3*). These genes from the vernalization and photoperiod pathways show complex interactions to promote flowering that are still not understood fully. In particular, *PPDH2 *function is assumed to be limited to the ability of a short photoperiod to promote flowering. Evidence from the fields of biodiversity, ecogeography, agronomy, and molecular genetics was combined to obtain a more complete overview of the potential role of *PPDH2 *in environmental adaptation in barley.

**Results:**

The dominant *PPDH2 *allele is represented widely in spring barley cultivars but is found only occasionally in modern winter cultivars that have strong vernalization requirements. However, old landraces from the Iberian Peninsula, which also have a vernalization requirement, possess this allele at a much higher frequency than modern winter barley cultivars. Under field conditions in which the vernalization requirement of winter cultivars is not satisfied, the dominant *PPDH2 *allele promotes flowering, even under increasing photoperiods above 12 h. This hypothesis was supported by expression analysis of vernalization-responsive genotypes. When the dominant allele of *PPDH2 *was expressed, this was associated with enhanced levels of *VRNH1 *and *VRNH3 *expression. Expression of these two genes is needed for the induction of flowering. Therefore, both in the field and under controlled conditions, *PPDH2 *has an effect of promotion of flowering.

**Conclusions:**

The dominant, ancestral, allele of *PPDH2 *is prevalent in southern European barley germplasm. The presence of the dominant allele is associated with early expression of *VRNH1 *and early flowering. We propose that *PPDH2 *promotes flowering of winter cultivars under all non-inductive conditions, i.e. under short days or long days in plants that have not satisfied their vernalization requirement. This mechanism is indicated to be a component of an adaptation syndrome of barley to Mediterranean conditions.

## Background

Temperature and photoperiod are the main environmental cues that regulate flowering time in winter cereals [1,2]. Barley (*Hordeum vulgare *L.) is classified as a long-day plant, which means that it will flower earlier when exposed to increasing day lengths. Some genotypes of barley require a period of prolonged exposure to cold during winter (vernalization) to accelerate the transition of the shoot apex from vegetative to reproductive development [3]. This combination of a requirement for vernalization and sensitivity to photoperiod ensures that flowering is postponed until after winter to avoid frost damage, but then occurs rapidly as day-length increases during spring, thereby avoiding heat and water stress during summer [4].

Wheat and barley cultivars are classified on the basis of their flowering behavior into two types of growth habit, namely winter and spring. The former requires prolonged exposure to low temperature to flower, whereas the latter group flowers rapidly without exposure to cold. Genetic studies have revealed that the epistatic relationships between three genes, *VRNH1*, *VRNH2*, and *VRNH3*, control the response to vernalization [5,6]. The winter growth habit depends on the combination of recessive alleles at *VRNH1 *and *VRNH3 *with the dominant allele at *VRNH2 *[5]. Genotypes that possess other allelic combinations for these genes exhibit a spring growth habit to different degrees. In agronomic classifications of barley germplasm, a third category of cultivars, termed facultative [7], is recognized, in which cultivars show winter hardiness but do not require vernalization.

The activity of *VRNH1 *is essential for flowering [8]. *VRNH1 *acts as a promoter of flowering, is induced by vernalization, and regulates the transition to the reproductive stage at the shoot apex [9]. Allelic variation at *VRNH1 *has been described, mainly in relation to deletions within the first intron [10-12]. These deletions are presumed to be responsible for the different vernalization requirements that are associated with different alleles. In plants that have not been vernalized, the deletions lead to differences in the levels of the *VRNH1 *transcript and, consequently, the allelic variation results in diverse flowering times [13,14].

*VRNH2 *is a floral repressor that delays flowering until plants are vernalized [5,15]. Allelic diversity at *VRNH2 *arises from the presence or deletion of a cluster of three genes (*ZCCT-H*) [7]. The null allele of *VRNH2 *corresponds to the recessive spring allele and is associated with rapid flowering [7,16,17]. Day length is the major determinant of the level of *VRNH2 *expression, with high levels of expression occurring during periods with long days [15,18,19].

*HvFT1*, candidate gene for *VRNH3*, is a homolog of the *FLOWERING LOCUS T *gene (*FT*) of *Arabidopsis thaliana *[20,21]. Strong evidence indicates that *VRNH3 *plays a central role in promoting flowering as an integrator of the vernalization and photoperiod pathways in temperate cereals [6,9,22]. Recently, novel *VRNH3 *alleles that show different adaptive effects have been identified by analyzing sequence polymorphisms and their phenotypic effects [23].

Two major photoperiod response genes, *PPDH1 *and *PPDH2*, have been reported in barley [1,24]. *PPDH1 *confers sensitivity to a long photoperiod and accelerates flowering under long days [25]. *HvFT3 *has been identified as a candidate gene for *PPDH2*, which is described as a gene that is responsive to a short photoperiod [21,22]. As far as can presently be determined, only two known *HvFT3 *(*PPDH2*) alleles exist, one of which is a null [14,31]. However, it is not possible to rule out that additional alleles are also present in cultivated barley. The dominant allele is the functional one, comprises four exons, and produces faster development towards flowering under short days. The recessive allele is a truncated gene, retaining only the 3' portion of exon 4 [22], and produces flowering delay under short days (Additional file 1).

The complexity and strength of the interactions reported among these genes indicate that they share the same regulatory network [26-28]. *VRNH1*, *VRNH2*, and *VRNH3 *form a feedback regulatory loop [6]. *VRNH1 *is probably the principal target of the vernalization signal [2]. Levels of the *VRNH2 *transcript are downregulated by short days and by a high level of *VRNH1 *expression [19]. Expression of *VRNH2 *delays flowering by inhibiting expression of *VRNH3 *[9]. After vernalization, transcription of *VRNH2 *decreases, which facilitates the upregulation of *VRNH3 *by long days in spring, and triggers flowering [4,6]. It is likely that the downregulation of *VRNH2 *is mediated by *VRNH1*. Photoperiod response genes also participate in the promotion to flowering. The dominant *PPDH1 *allele accelerates flowering by upregulating *VRNH3 *under long days [9]. *PPDH2 *is thought to upregulate *VRNH3 *expression under short-day conditions [22]. In addition, expression of *PPDH2 *has been detected both under short days [21,22] and under long days when the levels of *VRNH2 *transcript decrease [14].

As a result of these interactions, phenotypic responses of barley to environmental signals are complex. Natural allelic variation at these flowering time genes has been found in several studies in relation to responses to vernalization [9,11,29], photoperiod [21,30], or both [31,32]. This natural variation might be related to adaptation to different environmental conditions.

In the study reported herein, we investigated further the patterns of expression and interactions of *VRN *and *PPD *genes in a selection of vernalization-responsive barley cultivars. These cultivars represented different allelic combinations of *VRNH1, VRNH3*, and *PPDH2 *in a dominant *PPDH1 *and *VRNH2 *genetic background. The geographic distribution of *PPDH2 *alleles was analyzed in a wide array of barley germplasm that represented cultivars and landraces. In addition, the possible role of *PPDH2 *in the acceleration of flowering under long days was examined in a collection of winter cultivars, by analyzing their response to vernalization treatments of different duration.

## Results

### Distribution of *PPDH2 *alleles among domesticated barleys

We investigated the distribution of the *PPDH2 *alleles over a sample of 162 barley cultivars of different geographic origins (Additional file 2) and 159 Spanish landrace-derived inbred lines from the Spanish Barley Core Collection (SBCC) [33]. Lines were classified according to their seasonal growth habit, on the basis of the allelic constitution at *VRNH1 *and *VRNH2 *(Table 1). To enlarge the sample, we included previously published results for an additional 202 barley cultivars [21,31]. The dominant allele of *PPDH2 *gene was found in most of the spring cultivars (189 out of 206), whereas the majority of winter cultivars (102 out of 140) possessed the recessive (null) *ppdH2 *allele (Table 1). Facultative genotypes, characterized by having a winter allele at *VRNH1*, and a null allele (*vrnH2*) at *VRNH2*, did not show such a clear genetic distinction and approximately half (seven out of 18 cultivars) carried the dominant (functional) *PPDH2 *allele (Table 1). Strikingly, the allelic distribution among SBCC landraces differed from that observed in the commercial cultivars. Most of the winter Spanish landraces (127 out of 140) carried the functional *PPDH2 *allele (Table 1). The 140 winter SBCC landraces all carried the dominant allele at *VRNH2 *and *PPDH1 *but possessed two different alleles at *VRNH1*. According to the terminology for *VRNH1 *alleles proposed by Hemming et al. [13], 93 of these landraces carried *VRNH1-6 *and 47 carried the earlier flowering *VRNH1-4 *allele [14]. *PPDH2 *was carried at the same frequency among landraces carrying the *VRNH1-6 *and *VRNH1-4 *alleles. The wild-type recessive *VRNH1 *allele was not detected among the Spanish landraces.

**Table 1:** Distribution of *PPDH2 *alleles in barley cultivars and landraces of the Spanish Barley Core Collection (SBCC) classified according to their growth habit

	*Dominant *	*Recessive *
Commercial cultivars		
Spring	189	17
Faure et al. [21]	46	14
Cuesta-Marcos et al. [31]	82	2
Present study	61	1
Facultative	7	11
Cuesta-Marcos et al. [31]	3	3
Present study	4	8
Winter^a^	38	102
Faure et al. [21]	4	36
Cuesta-Marcos et al. [31]	4	8
Present study	30	58

SBCC landraces		
Spring	8	10
Facultative	1	0
Winter^a^	127	13

^a ^Winter lines include genotypes that carry *VRNH1-4*, *VRNH1-6 *or the wild-type *vrnh1 *allele at *VRNH1 *[12] and the dominant allele at *VRNH2*.

### Cold-induced gene expression under a long photoperiod

**Figure 1:**
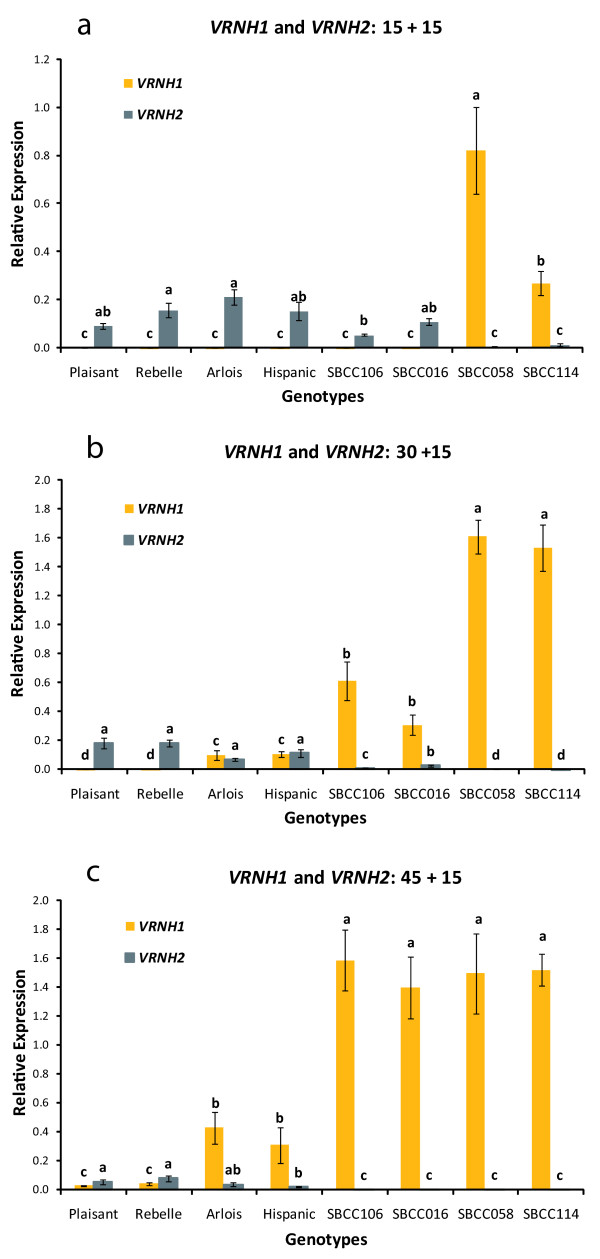
**Relative expression of *VRNH1 *and *VRNH2***. Detailed legend: Relative expression levels of *VRNH1 *and *VRNH2 *assayed by qRT-PCR in eight barley lines grown under a short photoperiod and different durations of vernalization: a) 15 d, b) 30 d, and c) 45 d. After vernalization, seedlings were subjected to no vernalization and a long photoperiod for 15 d. The results shown are normalized with respect to the level of the housekeeping gene *Actin *for each genotype and duration of vernalization. The variable of relative gene expression shown for each genotype and treatment is 2^ΔCT^, where ΔC_T _= C_T __Actin _- C_T __target gene_. Error bars represent the SEM. For each sampling time-point, bars with the same letter are not significantly different at P < 0.05 according to ANOVA that included all sampling time-points and genotypes per treatment.

**Figure 2:**
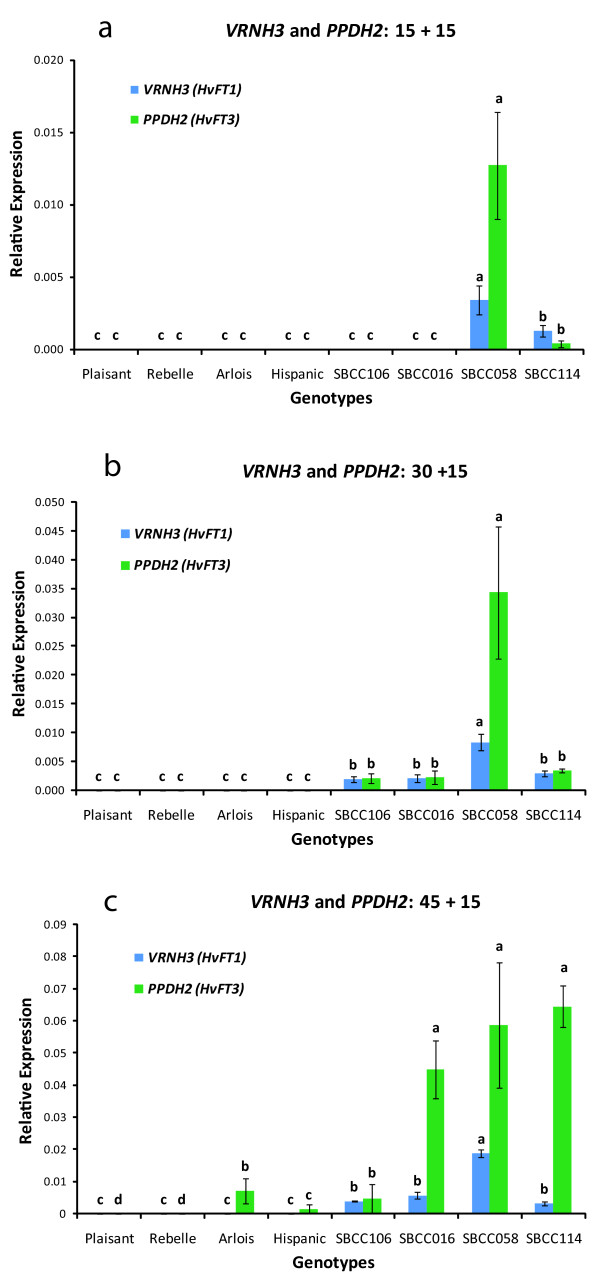
**Relative expression of *VRNH3 *and *PPDH2***. Detailed legend: Relative expression levels of *VRNH3 *and *PPDH2 *assayed by qRT-PCR in eight barley lines grown under different durations of vernalization and a short photoperiod: a) 15 d, b) 30 d, and c) 45 d. After vernalization, seedlings were subjected to no vernalization and a long photoperiod for 15 d. The results shown are normalized with respect to the level of the housekeeping gene *Actin *for each genotype and duration of vernalization. The variable of relative gene expression shown for each genotype and treatment is 2^ΔCT^, where ΔC_T _= C_T __Actin _- C_T __target gene_. Error bars represent the SEM. For each sampling time-point, bars with the same letter are not significantly different at P < 0.05 according to ANOVA that included all sampling time-points and genotypes per treatment.

Expression of the vernalization and photoperiod response genes was studied in eight barley lines, which represented four typical winter cultivars and four Spanish landraces (Table 2). The lines had been exposed to low temperature treatments of increasing length (15, 30 or 45 days) under short days, in every case ensued by growth for 15 days under long days (16 h light). All of the genotypes carried *VRNH2 *and the long-photoperiod-sensitive allele *PPDH1*, which allowed observation of possible interactions between these genes and the other vernalization and photoperiod genes. The expression profiles of the vernalization and photoperiod response genes were assessed by quantitative reverse-transcription PCR (qRT-PCR; Figures 1 and 2). Differences in expression among genotypes and treatments were found for *VRNH1*, *VRNH2*, *VRNH3*, and *PPDH2*. Although expression data for *PPDH1 *were also analyzed, its expression is not shown in Figures 1 and 2 because it was consistently high in all treatments and genotypes, and thus did not contribute to the variability of responses observed.

**Table 2:** Allelic configuration of genes associated with responses to vernalization and photoperiod in the genotypes selected for expression analysis

Vernalization and photoperiod genes								
**Genotype**	***VRNH1*^a^**	***VRNH2*^b^**	***VRNH3***				***PPDH1*^e^**	***PPDH2*^f^**
			**Promoter^c^**			**Intron 1^d^**		
					
			**SNP927**	**Indel 1**	**Indel 2**			

Plaisant	*vrnh1*	*VRNH2*	C	139	142	TC	*PPDH1*	*ppdH2*
Rebelle	*vrnh1*	*VRNH2*	C	135	146	AG	*PPDH1*	*ppdH2*
Arlois	*vrnh1*	*VRNH2*	C	139	142	TC	*PPDH1*	*PPDH2*
Hispanic	*vrnh1*	*VRNH2*	C	135	146	AG	*PPDH1*	*PPDH2*
SBCC106	*VRNH1-6*	*VRNH2*	C	139	142	TC	*PPDH1*	*PPDH2*
SBCC016	*VRNH1-6*	*VRNH2*	C	139	142	AG	*PPDH1*	*PPDH2*
SBCC058	*VRNH1-4 *	*VRNH2*	C	139	142	TC	*PPDH1*	*PPDH2*
SBCC114	*VRNH1-4*	*VRNH2*	C	139	142	AG	*PPDH1*	*PPDH2*

^a ^Alleles based on the size of intron 1 [12].

^b ^Presence/absence of *HvZCCT *[15].

^c ^Promoters identified previously [23].

^d ^Alleles based on two SNPs in intron 1, as reported previously [20].

^e ^Alleles based on SNP22 [25].

^f ^Presence/absence of *PPDH2 *[13].

In all genotypes, *VRNH1 *expression increased gradually with increasing duration of vernalization treatment, although differences in response between *VRNH1 *alleles were evident. After 15 d of cold treatment, *VRNH1 *expression was only detected in genotypes that carried the larger ~4 kb deletion in intron 1 (allele *VRNH1-4*), namely SBCC058 and SBCC114 (Figure 1a). The level of *VRNH1 *expression was significantly higher in SBCC058 than in SBCC114 (Figure 1a). After vernalization for 30 d, *VRNH1 *expression was detected in five genotypes (Figure 1b). *VRNH1 *expression was detected in all genotypes only after 45 d of cold treatment (Figure 1c). The expression level was highest for the *VRNH1-4 *and *VRNH1-6 *alleles (namely SBCC106 and SBCC016), and lowest for the wild-type recessive winter allele *vrnH1*, which was carried by Plaisant, Rebelle, Arlois, and Hispanic. Even though these four cultivars carried the same *VRNH1 *allele, they showed differences in *VRNH1 *expression (Figure 1b-c).

Although all lines carried the active *VRNH2 *allele, differences in its expression were observed (Figure 1a-c), and depended on the *VRNH1 *allele present. Of the four cultivars that carried the *vrnH1 *allele, expression of *VRNH2 *was much higher for Plaisant and Rebelle than for Arlois and Hispanic, with the exception of the shortest cold treatment (Figure 1a).

SBCC058 showed the highest level of *VRNH3 *expression under all conditions (Figure 2a-c). After 15 d of vernalization, *VRNH3 *was detected only in SBCC058 and SBCC114 (Figure 2a). *VRNH3 *expression was detected in SBCC106 and SBCC016 only after 30 d of cold treatment (Figure 2b). Under the experimental conditions used, *VRNH3 *expression was not detected in the four cultivars that carried the wild-type winter allele *vrnH1*.

Expression of *PPDH2 *was detected in all genotypes that carried the gene, i.e. all except Plaisant and Rebelle (Figure 2a-c). The level of *PPDH2 *expression increased with increasing duration of vernalization (Figure 2a-c), although the rate of increase differed among genotypes. After 15 d of cold treatment, only SBCC058 showed significant expression of *PPDH2*. In SBCC114, SBCC106, and SBCC016, *PPHD2 *expression was detected after 30 d of vernalization, but expression was not detected in Arlois or Hispanic until after 45 d of cold treatment (Figure 2a-c).

### Effect of *VRNH3*, *PPDH1*, and *PPDH2 *and different vernalization treatments on heading date in winter cultivars

To assess a possible effect of the major vernalization and photoperiod response genes on flowering time under natural conditions, we analyzed the time from planting to heading of 70 winter cultivars that were exposed to five different periods of vernalization, which ranged from 0 to 60 d, before transplantation to the field in Martonvásár, Hungary, on March 25^th^, which corresponds to a day length of 12 h 25 min. The list of barley lines and their genetic constitution for the major flowering-time genes is presented in Additional file 3. All of the lines carried the dominant allele at *VRNH2*. Although polymorphisms have been reported for the candidate genes (the *ZCCT-H *family), *VRNH2 *seems to be quite conserved among winter barleys, and just two alleles are usually assumed [12]. We evaluated the differences between the *PPDH1*, *PPDH2*, and *VRNH3 *alleles as a function of the duration of vernalization (Table 3). This was possible because there were enough individuals in each of the 8 classes resulting from the combination of the three genes to perform an analysis. Although the cultivars presented three different *VRNH1 *alleles (all showing a response to vernalization), they were so unevenly distributed over the sample (60 *vrnH1*, five *VRNH1-6*, five *VRNH1-4*) that it was not possible to include it as an additional factor in the analysis. All three genes analyzed showed significant effects on flowering time. On average, the dominant allele at *PPDH1 *accelerated the onset of flowering by 4 d. Lines that carried the functional allele at *PPDH2 *flowered 6 d earlier, and genotypes that carried the TC haplotype for *VRNH3 *flowered 2 d earlier. Consistent with the expectation for winter genotypes, different durations of vernalization had a significant effect on flowering time.

**Table 3:** Analysis of variance with REML of days to heading in the field after different vernalization treatments for 70 winter genotypes

Source of variation	df	ddf	F statistic	F pr
*PPDH1*	1	133	14.03	<0.001
*PPDH2*	1	133	22.07	<0.001
*VRNH3*	1	133	6.68	0.011
Vernalization treatment	4	544	169.68	<0.001

*PPDH1 *x *PPDH2*	1	133	0.45	0.504
*PPDH1 *x *VRNH3*	1	133	3.07	0.082
*PPDH2 *x *VRNH3*	1	133	0.07	0.793
*PPDH1 *x Ver treatment	4	544	2.57	0.037
*PPDH2 *x Ver treatment	4	544	7.88	<0.001
*VRNH3 *x Ver treatment	4	544	1.17	0.321

**Figure 3:**
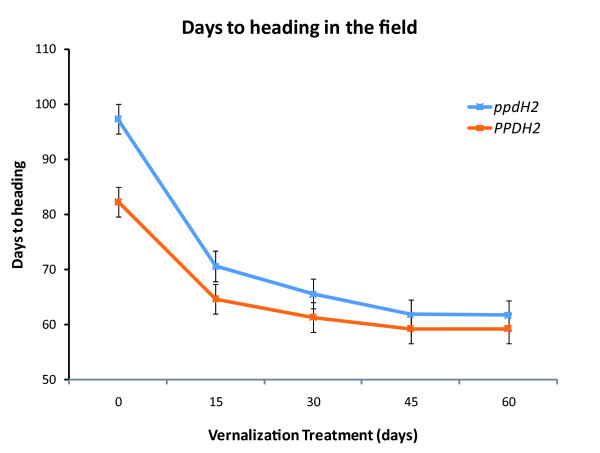
**Days to flowering in the field**. Detailed legend: Days to flowering of 70 winter cultivars planted on March 25th, 2010, after 0, 15, 30, 45 or 60 d of vernalization at 3°C under a 9-h light/15-h dark photoperiod with low light intensity. Orange - dominant allele (*PPDH2)*; blue - recessive allele (*ppdH2)*. Error bars represent the LSD (P < 0.05).

In the present analysis, a significant interaction between *PPDH2 *and the different cold treatments was detected (Figure 3). Exposure to a cold treatment before transplanting reduced the time to heading, although the reduction was not significant for vernalization treatments longer than 30 d. The presence of the dominant *PPDH2 *allele was associated with earlier flowering in plants that had not been vernalized fully (0 or 15 d cold treatment; Figure 3).

**Figure 4:**
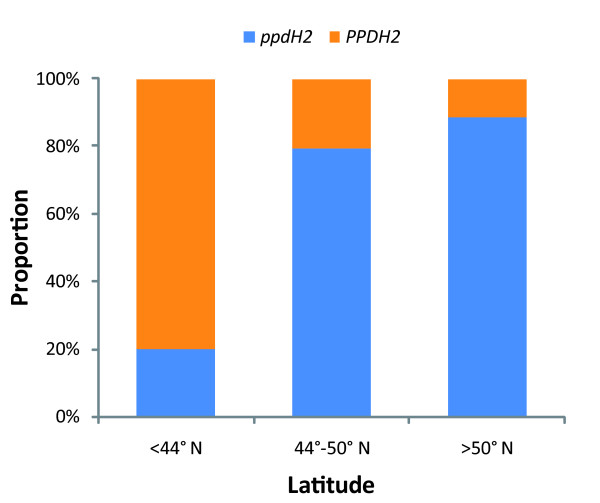
**Distribution of *PPDH2 *in winter cultivars**. Distribution of *PPDH2 *alleles in 125 winter barley cultivars classified according to latitude of origin. Orange - dominant allele (*PPDH2)*; blue - recessive allele (*ppdH2)*.

We also analyzed the geographic distribution of 125 winter barley cultivars, which were assigned to their predicted phenotype on the basis of the presence of a complete *HvFT3 *gene, and classified into three classes according to latitude. The *PPDH2 *dominant allele was predominant in winter cultivars from southern latitudes, whereas the proportion of cultivars with the recessive (null) allele *ppdH2 *was greater at higher latitudes (Figure 4).

## Discussion

Heading date is a crucial trait for the adaptation of barley to different areas of cultivation and cropping seasons. Traditionally, cultivars are classified into spring, facultative, and winter types on the basis of their flowering habit. This is an agronomic classification that is based on phenotypic behavior. It is a useful simplification that summarizes a more complex and diverse array of responses at the genetic level. Study of the genes involved in the photoperiod and vernalization pathways in cereals is continuously producing new information that is shedding light on the nature of adaptation of cultivars and on the variety of phenotypic responses produced by the combination of photoperiod and vernalization genes carried by individual cultivars.

### *PPDH2 *is not distributed randomly in barley germplasm

The spread of cultivated barley out of its area of origin was driven by the occurrence of phenotypic variation that resulted from the appearance of new multilocus flowering-time haplotypes at *VRNH1*, *VRNH2*, *PPDH1*, and *PPDH2 *[32]. Mutations in *VRNH1 *allowed the expansion of cultivated barley from midlatitudinal regions to lower and higher latitudes, where spring types are common [29,32,34]. The entry of barley to Europe occurred via several routes [34]; One of them, to the North and then West, via the Balkan Peninsula, and another one towards the Southwest, then through North Africa, reaching Europe through Spain. In the first case the environmental conditions (long winters, shorter days than in the Mediterranean region) favoured the recessive allele in *PPDH2 *so its frequency increased significantly within the winter forms. In the latter case, the ancestral form was not selected out from the winter barleys, which is exactly the case for the Spanish landraces.

In midlatitudinal regions, including North Africa, southern Europe, Nepal, China, and Japan, both spring and winter barley types are cultivated. However, in these regions, the dichotomic agronomic classification is insufficient to describe the range of vernalization responses found, in which *VRNH1 *plays a central role. Allelic diversity at *VRNH1 *has been described by several authors [11-14]. This diversity is the result of deletions or insertions within the first intron of the gene, and is associated with a gradation of vernalization responses from strict winter to spring types. In general, the larger the deletion, the shorter the vernalization period required.

Although, originally, wild barley carried the photoperiod-responsive alleles *PPDH1 *and *PPDH2 *(dominant allele), mutant, nonresponsive alleles of these genes originated before domestication [32]. The appearance of the nonresponsive *ppdh1 *allele allowed the cultivation of barley to spread to more northerly regions [30]. Regarding *PPDH2*, some authors have already pointed out the prevalence of the dominant allele in spring cultivars, and its relative scarcity in winter cultivars [21,31]. In the present study, exclusively done with winter types, we found that, the dominant *PPDH2 *allele was frequent at lower latitudes (<44°N) but not at higher latitudes. The dominant allele was also prevalent in a large set of winter landraces cultivated on the Iberian Peninsula (35-44°N). This pattern is remarkable, because latitudes below 44°N include almost the entire Mediterranean region. In this region, barley is sown predominantly during autumn and, to a large extent, using winter cultivars.

The adaptive role of *PPDH2 *is confirmed by its influence on key agronomic traits. It was identified originally as a short-photoperiod quantitative trait locus in winter × spring barley crosses [1,35]. Its effect is especially large in Mediterranean latitudes, where it has been identified as the main QTL that affects flowering, together with *Eam6 *[35,36]. It also affects grain yield indirectly, through flowering date, under Mediterranean conditions [37].

### *PPDH2 *expression is mediated by the vernalization pathway in winter cultivars

Analysis of gene expression can provide indications of the role of *PPDH2 *and interacting genes. To be meaningful for the Mediterranean region, we chose to carry out this study with winter genotypes, unlike previous studies [21,22] which focused on the effect of *PPDH2 *on spring genotypes.

Expression of photoperiod and vernalization response genes show strong interactions [6,26,28]. A long photoperiod induces *VRNH2 *expression [19], which then represses expression of *VRNH3 *[9] and *PPDH2 *[14]. The model currently accepted proposes that during autumn and winter (low temperature and short days), vernalization induces *VRNH1 *expression and the short photoperiod downregulates *VRNH2 *expression [2,19]. Subsequently, in spring, *VRNH1 *is relatively high, much more rapidly if vernalization was sufficient. Although the long photoperiod conditions in spring are favorable for *VRNH2 *expression, *VRNH2 *is repressed by the expression of *VRNH1*. Once the vernalization requirement has been satisfied, *VRNH3 *expression is induced by long days [9], after which the plants are committed irreversibly to reproductive development.

In our expression analysis, we compared three different *VRNH1 *alleles. At each time-point examined, the expression level was lower in the four winter cultivars that carried the full-length intron than in the four SBCC lines that carried two different deletions. As proposed previously [19], vernalization did not block the induction of *VRNH2 *in response to increasing day length, which was detected under long days after 15 or 30 d of cold treatment. Once *VRNH1 *is expressed, it can then begin to repress *VRNH2 *expression. However, the differences in responses observed among the four winter cultivars that carried the strict winter allele at *VRNH1 *were unexpected. Two of these cultivars (Plaisant and Rebelle) behaved as expected; a long period (45 d) of cold induction was needed to detect *VRNH1 *expression. Interestingly, for the other two cultivars (Arlois and Hispanic), we detected expression of *VRNH1 *after only 30 d of cold treatment, and the transcript level increased further after 45 d of treatment. These four cultivars carry identical, recessive alleles at *VRNH1 *and *VRNH3*, and dominant alleles at *VRNH2 *and *PPDH1*. Among the genes investigated, they differ only at *PPDH2*, which leads us to think of a possible role of this gene in the earlier induction of *VRNH1 *expression. However, we cannot rule out the possibility that additional genes might be responsible for this induction.

In a previous study, we did not detect *VRNH3 *expression in some of these genotypes when they were grown without vernalization under a long photoperiod (SBCC058 and Plaisant) or vernalized under a short photoperiod (SBCC058, SBCC106 and Plaisant) [14]. In winter genotypes, a period of cold induction is required before *VRNH3 *expression can be induced by long days, as reported already for the wild-type *vrnH1 *winter allele [9]. In the present study, we included cultivars that represented several recessive alleles at *VRNH3*, because we had previous evidence that they might produce differences in heading date in the field among these cultivars [23]. Different expression between *VRNH3 *alleles was detected only for the pair of lines with the largest deletion in *VRNH1 *(SBCC058 and SBCC114). The TC allele showed higher expression than the AG allele, in the same direction as reported in a previous study [23]. However, there was no difference in *VRNH3 *expression between SBCC016 and SBCC106, which showed the same polymorphism at *VRNH3 *among them than SBCC114 and SBCC058. Either the duration of the experiment was insufficient to reveal possible differences or other genes that are unaccounted for at present influence this pathway.

Although the expression of *PPDH2 *is higher under a short photoperiod, we and other authors [14,22] have reported *PPDH2 *expression under a long photoperiod. Expression of *PPDH2 *was detected at some time -point in all genotypes that carried the dominant allele of *PPDH2*, irrespective of day length. In winter genotypes, *VRNH2 *must be absent or clearly receding (either because lack of induction under short days, or repression by expression of *VRNH1*) for *PPDH2 *to be expressed.

### *PPDH2 *promotes flowering irrespective of photoperiod under noninductive conditions

An additional question concerns the nature of the role of *PPDH2*. *PPDH2 *has been suggested to affect the promotion of the transition of the shoot apical meristem from vegetative to reproductive, in the end affecting flowering. The two experiments that support this hypothesis, however, propose different modes of action for *PPDH2*. On one hand [21], it was proposed that *HvFT3 *(*PPDH2*) substituted *HvFT1 *(*VRNH3*) as the trigger to induce flowering under short days (8 h), although its expression was not sufficient to induce the transition to the reproductive stage. They did not find *HvFT1 *(*VRNH3*) induction with 8 h of light, even after the transition of the meristem had taken place. Another study [22], concluded that *HvFT3 *acts as a floral promoter under short days (12 h this time), but through the induction of *HvFT1 *(*VRNH3*). Therefore, it seems proven that *PPDH2 *promotes flowering under short days, but the mechanism (or mechanisms) of action are not clear yet. The experiments just reported used different genotypes and, probably more important, different day lengths. Differences in induction of genes may have been caused by different critical day length thresholds needed for expression of these genes. In any case, all the genotypes tested in those studies were spring lines, and the interaction of *PPDH2 *with the vernalization pathway in winter genotypes, at gene expression level, remained largely unexplored.

By investigating simultaneously the expression of the flowering response genes, we observed that *VRNH1 *and *PPDH2 *were expressed before *VRNH3 *in all six vernalization-responsive genotypes tested. Our results agree with a comparative model proposed by Higgins et al. [28]. In that scheme, *PPDH2 *promotes *VRNH1 *expression under short-day conditions. We propose that *PPDH2 *has a more general role for winter cultivars, and promotes flowering under all noninductive conditions, i.e. under short days or long days in plants that have not satisfied their vernalization requirement.

This hypothesis is supported by the field trial observations. Heading date in our trial occurred from May 10^th ^until July 13^th^. The photoperiod experienced by the plants increased from 12 h 25 min at transplanting to 14 h 53 min when the first genotype reached heading, and then kept increasing until 15 h 58 min on June 21^st^. Therefore, most of the growth period of the plants occurred in photoperiods well above 12 h. We observed a concurrent effect of *PPDH1 *and *PPDH2 *on flowering, which agrees with the concurrent effect for these two genes found under a 12 h photoperiod [38]. During this period of the year (May-July), and even earlier, the effect of long days on heading date in experiments carried out in temperate latitudes can be detected through its effect on *PPDH1 *[39].

Heading date was distinctly earlier for winter genotypes that carried the dominant *PPDH2 *allele than for cultivars that possessed the recessive allele. The difference was especially marked for plants that had not been vernalized or had experienced only a short cold period. The 70 genotypes used might show some intrinsic difference in earliness *per se *that might account for some of the differences that could be attributed to *PPDH2 *as the main factor. However, the differences in heading that were caused by *PPDH2 *decreased gradually as the duration of vernalization increased. This interaction between *PPDH2 *and duration of vernalization treatment was quite reliable, and is consistent with the role for *PPDH2 *suggested above. Other authors [40] have also reported an effect of *PPDH2 *on flowering time under long photoperiods, but only with the application of synchronous photo and thermo cycles, and when specific allelic configurations are present at the *PPDH1 *and *VRNH1 *loci.

Winter genotypes are cultivated normally in areas where they are exposed to sufficient vernalization during winter. As a consequence, these genotypes do not need to express other genes that promote flowering. By contrast, in spring cultivars, *PPDH2 *can facilitate flowering and ensure timely completion of such a short vital cycle. However, in winter cultivars with lower requirements for vernalization, such as those adapted to geographical areas with traditionally mild winters, as exemplified by Mediterranean climates, the presence of *PPDH2 *might help to induce flowering when the vernalization requirement has not been satisfied fully (which is a not unusual phenomenon under natural conditions in the Iberian Peninsula). This could explain why the majority of SBCC winter lines carry the dominant *PPDH2 *allele. SBCC winter lines are adapted to a typical mild Mediterranean winter, in which temperatures are not very low. If the cold period is insufficiently long to satisfy the vernalization requirement of these genotypes, *PPDH2 *could act as a compensatory mechanism to accelerate flowering and ensure it occurs at the optimal time, possibly before the effect of a sensitive *PPDH1 *is noticeable. In some barley and wheat cultivars the vernalization requirement can be replaced, partially or completely by exposure to short photoperiods [18,41,42]. This phenomenon, known as short-day vernalization [42] has been reported in barley genotypes with winter alleles in *VRNH1 *and *VRNH2 *and dominant *PPDH2 *[1,35]. In these genotypes, a dual short day-long day induction of flowering could take place [18]. This dual mechanism is present in many species, including many *Festucoideae *[43]. King and Heide [43], proposed that "...as an evolutionary mechanism, the versatility of the alternative short day/vernalization primary induction system offers a beautiful safety mechanism with short days acting as a fall-back alternative in case of inadequate winter chill". The involvement of *VRN2 *in the genetic basis of this mechanism was already put forward by Dubcovsky et al. [18], because "the convergence of photoperiod and vernalization signals at the *VRN2 *gene, provides a possible explanation to the interchangeability of short day and vernalization treatments."

The presence of the dominant *PPDH2 *allele would not be necessary under conditions in which vernalization occurred inevitably year after year, as it is common in more northerly latitudes. Actually, other authors have claimed that the presence of the dominant allele at *PPDH2 *is not a desirable feature for winter barley [44,45], because it would induce progress towards flowering too early [21], with undesirable agronomic consequences, including loss of frost tolerance. This may well be true for strict winter cultivars (strict winter *vrnH1 *allele plus dominant *VRNH2*) in more northerly latitudes. The null, late-flowering allele would be more suitable for an autumn-sown cultivar because it would keep plants in the vegetative growth phase longer [46], perhaps through maintaining the expression of genes that confer tolerance to low temperature [47]. On the basis of these studies, negative agronomic effects of the dominant *PPDH2 *allele should be investigated, especially in relation to freezing tolerance. However, a dominant *PPDH2 *allele could be a good option for cultivars cultivated in geographic areas where the winters are not that cold. The adaptation syndrome for barley landraces in the Iberian Peninsula seems to be the combination of an appropriate *VRNH1 *allele with dominant *PPDH1*, to ensure that flowering will occur before temperature rises too high, and with a dominant *PPDH2 *to ensure that plant growth will be not too delayed even in the years that conditions do not produce full vernalization.

## Conclusions

It is crucial to study the main genes involved in the vernalization and photoperiod pathways simultaneously, because this enables the interactions and functions of these genes to be interpreted more accurately, and their involvement in the induction of flowering to be elucidated.

There is a wide agreement over the central role of *VRNH1 *on the control of the progress of barley towards flowering. Nevertheless, different flowering-time responses seem to be modulated by the alleles present at the other vernalization and photoperiod genes *VRNH2*, *VRNH3*, *PPDH1*, and *PPDH2*. Of these genes, *PPDH2 *might have an important role in the regulation of *VRNH1*, especially under a long photoperiod, by upregulating *VRNH1 *expression and indirectly reducing the time to flower.

*PPDH2 *has a strong effect on heading date in a wide array of winter genotypes. The dominant allele at *PPDH2 *accelerates flowering under long days in plants in which the vernalization requirement has not been satisfied. The presence of *PPDH2 *in most winter landrace-derived lines of the SBCC indicates this allele could promote adaptation to geographic areas with milder winters, such as Mediterranean environments.

We also suggest the *PPDH2-*dependent mechanism proposed in this study could be complementary to the mechanism governed by *PPDH1*. The sensitive *PPDH1 *allele is typical of winter cultivars and *PPDH2 *is more common in spring cultivars. Both mechanisms promote flowering in different environments. Furthermore, in Mediterranean environments, these two mechanisms could be combined to facilitate flowering in optimal conditions.

## Methods

### Genotyping

A set of 162 barley genotypes (Additional file 1) and 159 landraces from the SBCC were genotyped for the vernalization (*VRNH1*, *VRNH2*, and *VRNH3*) and photoperiod (*PPDH1 *and *PPDH2*) genes as described previously [14,23]. Genotyping was conducted on single plants of each accession, partly at ARI-HAS (Hungary) and partly at EEAD-CSIC (Spain).

### Gene expression analysis

#### Plant material

Eight winter genotypes of barley were chosen to assess differences in the expression of the five major genes involved in responses to temperature and photoperiod. The genotypes consisted of the French cultivars Rebelle ((Barbarrosa × Monarca) × Pirate), Plaisant (Ager × Nymphe), Hispanic (Mosar × (Flika × Lada)), and Arlois (unknown pedigree), and four inbred lines, derived from landraces, that belong to the SBCC [33]. The genotypes studied have different *VRNH1*-*VRNH3 *allelic combinations and all can be classified as 'winter' genotypes. The genotypes could be grouped into four pairs, with each pair sharing the same *VRNH1 *allele, as defined by the length of the first intron. Each pair defined on the basis of *VRNH1 *structure was polymorphic for *VRNH3 *(Table 2), as defined by single nucleotide polymorphisms (SNPs) in intron 1, as reported previously [20], and by indels in the promoter region [23]. All genotypes carried an active *VRNH2 *and the sensitive allele at *PPDH1*, and all carried the *PPDH2 *functional allele except Rebelle and Plaisant (Table 2).

#### Conditions of plant growth

For expression studies, seeds of the eight genotypes were sown in pots and germinated in a sunlit glasshouse at 19 ± 1°C with a 16-h light/8-h dark photoperiod. Ten days after sowing, when the plants had reached the two-leaf stage (Z12, Zadoks scale [48]), the seedlings were moved to a growth chamber and exposed to 7 ± 1°C for 15, 30 or 45 d under a short photoperiod (8-h light/16-h dark) and low light intensity (12 μmol m^-2 ^s^-1^). After vernalization, the plants were transferred sequentially to an additional growth chamber maintained at 22 ± 1°C under a 16-h light/8-h dark photoperiod with light intensity of 220 μmol m^-2 ^s^-1^, where they were kept for 15 d, after which whole seedlings, excluding root tissue, were harvested. Harvesting took place in the middle of the light period. Four individual plants were harvested per sampling time-point and genotype, and were treated as four biological replicates.

#### RT-PCR and real-time PCR analysis

Extraction of RNA and preparation of cDNA followed the methods reported previously [14]. qRT-PCR was performed for all of the samples harvested. Amplifications were carried out in 20-μl reactions that contained 10 μl of SYBR Green Quantimix Easy SYG Kit (Biotools, Madrid, Spain), 0.3 μM each primer, 4 mM MgCl_2_, and 4 μl of cDNA, which corresponded to 300 ng of total RNA. Reactions were run on an ABI7500 real-time PCR system (Applied Biosystems). Cycling conditions for *VRNH1*, *VRNH2*, *VRNH3*, *Actin*, and *PPDH2 *were 6 min at 95°C, followed by 40 cycles of 15 s at 95°C, 15 s at 60°C, and 50 s at 72°C, and this was followed immediately by a melting curve program (60-95°C). Fluorescence data were acquired during the elongation step at 72°C and during the melting curve program. Two identical reactions (technical repeats) were performed per sample for each cDNA-primer combination. Levels of *Actin *expression were also quantified in the same run as an internal control. Four biological repeats were analyzed and showed similar trends. Expression levels were calculated using the ABI 7500 software package (Applied Biosystems). Gene expression at each time-point was normalized to the expression of *Actin*.

#### Statistical analysis of differences in gene expression

Differences in relative expression between genotypes and treatments were evaluated using the analysis of variance (ANOVA) procedure in SAS [49]. The variable used for the analysis of each treatment and genotype was ΔC_T _(C_T _actin - C_T _target gene). This variable was preferred over the more commonly used 2^-ΔCT ^because of the concerns expressed regarding its use for statistical analysis [50]. The ANOVA model included biological replication, genotype, treatments, and genotype-by-treatment interactions. Genotypes and treatments were considered as fixed factors. The variability that resulted from biological repeats and their interaction with the other factors was used as the error term to test genotype and treatment, as well as their interaction. A multiple means separation was carried out using the least significant difference (LSD) test (P < 0.05) for the main effects that were significant in the ANOVA. Each value included in the analysis was the average of two technical repeats to protect against slight fluctuations in reading and small pipetting errors.

#### Field trial of winter cultivars after vernalization treatment

Sensitivity to vernalization and the subsequent flowering behavior of a set of 70 winter barley genotypes (Additional file 3) were evaluated. A vernalization period was imposed using the Martonvásár Phytotron (Hungary), in accordance with procedures described previously [51]. Vernalization was applied in 15-day increments, to give a total of five treatments that ranged from no vernalization to 60 d of vernalization at a temperature of 3°C, under an 8-h light/16-h dark photoperiod and low light intensity (12-13 μmol m^-2 ^s^-1^). After vernalization, seedlings were transplanted by hand to the field at Martonvásár, Hungary, on March 25^th^, 2010, when the average photoperiod was 12 h. Two plants were evaluated per genotype and treatment. For each plant, the number of days to flowering (Z49, Zadoks scale [48]) was scored. The trial was terminated after 100 d. Plants that had not headed were given a value of 150 d to heading.

### Statistical analysis of field trials

Differences in days to heading were analyzed by means of ANOVA. *VRNH3*, *PPDH1*, and *PPDH2 *and vernalization treatment were included as fixed main factors. Replications were nested into genotypes. ANOVA was performed in Genstat 13 (VSN International, Hemel Hempstead, UK), using restricted maximum likelihood (REML) to account for the unequal number of units in each cell. Two-way interactions were also included in the model. Given that all winter cultivars carry allelic combinations for the winter growth habit at *VRNH1 *and *VRNH2*, these two genes were not considered in the statistical analysis. All *VRNH1 *alleles were pooled, because the population was very unbalanced with respect to this locus (60 genotypes carried the recessive *vrnH1 *allele, five possessed *VRNH1-4*, and five contained *VRNH1-6*) and inclusion of this factor meant that the analysis could not be performed.

## Authors' contributions

The idea for the manuscript arose in discussions between AMC, MCC, EI, and IK. MCC, EI, and AMC conceived the gene expression study and participated in its design; IK and OV devised and conducted the field experiment; MCC analyzed gene expression; IK and AMC carried out genotyping and proposed the latitudinal analysis; IK scored the phenotypes in the field experiment; EI performed the statistical analyses; MPG and OV provided interpretations of the results to place them in an agronomic context; MCC drafted the manuscript; IK, EI, and AMC carried out thorough revisions of the draft. All authors read and approved the final manuscript.

## Supplementary Material

Additional file 1 **Short day sensitivity**. Description of the nature and function of the *PPDH2 *alleles, compared to previous reports in the literature. Click here for file

Additional file 2 **Barley cultivars characterized in this study**. The country of origin, row number, and alleles present at *VRNH1*, *VRNH2*, *VRNH3*, *PPDH1*, and *PPDH2 *are presented. Click here for file

Additional file 3 **Winter barley cultivars included in the field trial**. The country of origin, row number, growth habit, alleles present at *VRNH1*, *VRNH2*, *VRNH3*, *PPDH1*, and *PPDH2*, and the sources of the information are presented. Click here for file
