# Supplementary material for: Adaptation of barley to mild winters: A role for PPDH2
Source: BMC Plant Biol. 2011 Nov 18;11:164. doi: 10.1186/1471-2229-11-164 (PMC3226555; doi:10.1186/1471-2229-11-164)
Supplement: Additional file 1 — Short day sensitivity. Description of the nature and function of the PPDH2 alleles, compared to previous reports in the literature. [file 1471-2229-11-164-S1.DOC]

**Sensitivity to short photoperiod**

Sensitivity of barley to short photoperiod has been described in two different ways. Both views acknowledge the role of *PPDH2* in short day sensitivity. For one school of thought, the sensitive allele is the recessive one, because the absence of the functional allele hinders the development of the plant, which stays vegetative for a longer time under short days. This view was first expressed by Mahfoozi et al [1], and then maintained in many works by authors linked to this group. For the other school, the presence of the functional allele stimulates growth under short days and, therefore, genotypes with the dominant PPDH2 allele are named short day-responsive [2,3,4]. Though we have not found a specific definition of sensitivity in articles representing this second view, it is clearly implied in their works that the sensitive genotypes are those whose growth is promoted under short days. Both views agree in declaring the recessive *PPDH2* allele as the null one, and as the responsible for delayed heading under short days (whereas the opposite is true for the dominant one). The only difference between these two views lies in what they name “sensitive” and “insensitive”.

In contrast to the confusing situation with short days and *PPDH2*, there is consensus on how to describe sensitivity to long photoperiod, mainly governed by *PPDH1* in barley: the sensitive allele is the one that shortens the plant cycle when subjected to long days. In this case, the physiological and biochemical mechanisms underlying the phenomenon are well-known, and support the definition as it stands. There is also consensus on how to define sensitivity to cold temperatures (vernalization). Sensitive cultivars are those that experience faster promotion towards flowering after a prolonged period of vegetative growth under cold temperatures. In this case, the underlying physiological and biochemical mechanisms are also well-known.

It seems that the two ways of describing sensitivity to short days could be derived from which of these two better-known phenomena was taken as a model. Short days usually occur concurrently with vernalizing temperatures and thus, it makes sense to draw a parallel between the sensitivity to these two environmental stimuli. Therefore, according to this view, “sensitive” would mean “the allele that delays flowering”. On the other hand, it also seems intuitive to treat both sensitivities to photoperiod (long and short) in the same way, i.e., to name “sensitive” to the allele that induces earlier flowering. This view finds physiological justification in the hypothesis of dual induction (short days, long days), that acts in some genotypes of wheat and barley, among other species, well summarized by Dubcovsky et al. [3] and Faure et al. [5]. Therefore, both views have some justification.

According to Merriam-Webster, “sensitive” means “capable of being stimulated or excited by external agents”. Following this authority, it seems more intuitive to declare the dominant allele “sensitive”, as it is the one that performs an action (gene expression with an effect on phenotype) in response to stimulus. On the contrary, the null allele produces a truncated protein which is not functional, and has no effect on the plant. The problem is, we do not know for sure which stimulus this is. Short days have long been recognized as the stimulus inducing *PPDH2* effect. But, how short are short days? The expression and phenotypic effect of *PPDH2* dominant allele have been found under different day length conditions. Expression seems maximal under photoperiods from 8 h to 12 h (see main text and references therein), but it has also been found (though at a lower level) under 16 h light. A phenotypic effect of *PPDH2* has been found under photoperiods below 12 h, but also at 12 h [4] and, in this manuscript, clearly above 12 h. The fact that *PPDH2* expression is likely repressed by *VRNH2*, which is expressed under long days, produces an apparent response to short days in winter cultivars. This is one the reasons that has precluded the detection of any effect of *PPDH2* under long days. But the stimulus that induces expression of *PPDH2* remains largely unknown. For this reason, we prefer not to declare any of its alleles as “sensitive”. Sensitive to what? We believe it is better to leave the “sensitive/insensitive” question open, until this last question is finally solved.

**References**

1. Mahfoozi S, Limin, AE, Hayes PM, Hucl P, Fowler DB: Influence of photoperiod response on the expression of cold hardiness in wheat and barley. *Can J Plant Sci* 2000, 80: 721–724

2. Roberts EH, Summerfield RJ, Cooper JP, Ellis RH: **Environmental control of flowering in barley (*Hordeum vulgare* L.). I. Photoperiod limits to long-day responses, photoperiod-insensitive phases and effects of low-temperature and short-day vernalization**. *Ann Bot-London* 1988, **62**:127-144.

3. Dubcovsky J, Loukoianov A, Fu D, Valarik M, Sanchez A, Yan L: **Effect of photoperiod on the regulation of wheat vernalization genes VRN1 and VRN2**. *Plant Mol Biol* 2006, **60**:469-480.

4. Sameri M, Pourkheirandish M, Chen G, Tonooka T, Komatsuda T: **Detection of photoperiod responsive and non-responsive flowering time QTL in barley**. *Breeding Sci* 2011, **61**:183-188.

5. Faure S, Higgins J, Turner A, Laurie DA: **The FLOWERING LOCUS T-like gene family in barley (*Hordeum vulgare*)**. *Genetics* 2007, **176**:599-609.
